# The Effect of High Velocity Low Amplitude Cervical Manipulations on the Musculoskeletal System: Literature Review

**DOI:** 10.7759/cureus.7682

**Published:** 2020-04-15

**Authors:** Andrea Giacalone, Massimiliano Febbi, Fabrizio Magnifica, Enzo Ruberti

**Affiliations:** 1 Industrial Engineering Technologies for Sports Medicine and Rehabilitation, University of Rome Tor Vergata, Rome, ITA; 2 Neurology, Sapienza University, Rome, ITA; 3 Aerospace Medicine, Diagnostic Therapeutic and Rehabilitative Aeromedical Center, Italian Air Force, Rome, ITA

**Keywords:** cervical manipulation, neck pain, osteopathic, pressure pain threshold, hvla, thrust techniques

## Abstract

In manual therapy, high velocity low amplitude (HVLA) cervical manipulation techniques are frequently used, but often the physiological and biomechanical effects that can be obtained are not completely clear. The techniques are mostly used for the treatment of biomechanical joint dysfunction, but little is yet known about the possibility of using them in order to achieve better performance on healthy subjects. The objective of the study is to describe how cervical manipulation can impact on a musculoskeletal disorder.

A systematic search was carried out on the Pubmed electronic database from the beginning of January to March 2020. Two independent reviewers conducted the screening process through the PRISMA diagram to determine the eligibility of the articles. The inclusion criteria covered randomized controlled trial (RCT) manuscripts published in peer-reviewed journals with individuals of all ages from 2005 to 2020. The included intervention was thrust manipulation or HVLA directed towards the cervical spine region. After reviewing the literature, 21 of 74 articles were considered useful and relevant to the research question.

The results of the research show that HVLA techniques, on subjects with musculoskeletal disorders, are able to influence pain modulation, mobility and strength both in the treated area and at a distance. Cervical manipulations are effective in management of cervicalgia, epicondylalgia, temporomandibular joint disorders and shoulder pain. With regard to results on strength in healthy subjects, given the divergent opinions of the authors, we cannot yet state that manipulation can significantly influence this parameter.

Cervical manipulations can also have risks for the patient if applied when not appropriate but the frequency of complications due to vertebral manipulation are very low. However, the manipulation techniques might be limited by low patients tolerance or the presence of contraindications. In addition, the optimal number of manipulations to be performed and the long-term benefits produced are unknown.

## Introduction and background

Neck pain is a musculoskeletal symptom associated with disability and significant economic health costs. Neck pain has been classified as one of the top two largest reasons for disability caused by musculoskeletal pain conditions by the Global Burden of Disease studies. It has been reported that 70% of the general population will experience neck pain at some time during their lives; however, the global point prevalence is 4.9% [1-3].

The number of people suffering from skeletal muscle problems is constantly increasing, often due to work activities that lead us to assume the wrong position for a prolonged period of time. The symptoms that are perceived by the subject can be various: stiffness, pain in the cervical area and muscles associated with it, tingling along the upper limbs, loss of strength, brachialgia, headaches and dizziness [4-6].

Spinal manipulation is a manual therapy technique used by chiropractors, osteopaths, physiotherapists and some doctors to treat skeletal muscle problems.

The use of cervical manipulation has reported positive results on pain reduction, cervical mobility, and general function in subjects with non-specific mechanical cervical pain.

Vertebral manipulations have been shown to produce different effects on our body including: increased strength, changes in somatic and visceral reflexes, central cortical neuron processing and cortical motor control of upper extremity muscles, sensory-motor integration and can also produce changes in nociceptive pain by increasing the pressure pain threshold. These sensory inputs can influence efferent pathways in the cervical spine by modifying the excitability of the alpha motor neuron, with subsequent changes in the level of muscle activity [7-9].

The use of high velocity low amplitude (HVLA) techniques is growing as a therapeutic option, the review in question aims to provide a general overview of the effects they can produce.

## Review

Materials and methods

The method used to conduct this research was the selection of articles related to a population study of subjects with skeletal muscle or healthy problems (Population), to evaluate the effects of cervical manipulation interventions (Intervention), comparing them with control subjects receiving placebo or another type of treatment (Control), resulting in the outcomes produced by authors of selected studies (Outcomes).

Research Strategy

P (population): healthy people or people with skeletal muscle problems

I (intervention): HVLA cervical manipulations

C (comparison): patients receiving placebo or another type of treatment

O (outcome): improved symptomatology or strength

Research question: What effects can HVLA cervical manipulation techniques have on subjects suffering from musculo-skeletal disorders or on healthy subjects?

A literature search was carried out on the Pubmed search engine, with the following keywords, using the Mesh thesaurus and Bolean operators: "Cervical Thrust Manipulation"[Mesh] OR "Cervical HVLA"[Mesh] AND "Effects"[Mesh] AND "last 15 years"[PDat].

**Figure 1:**
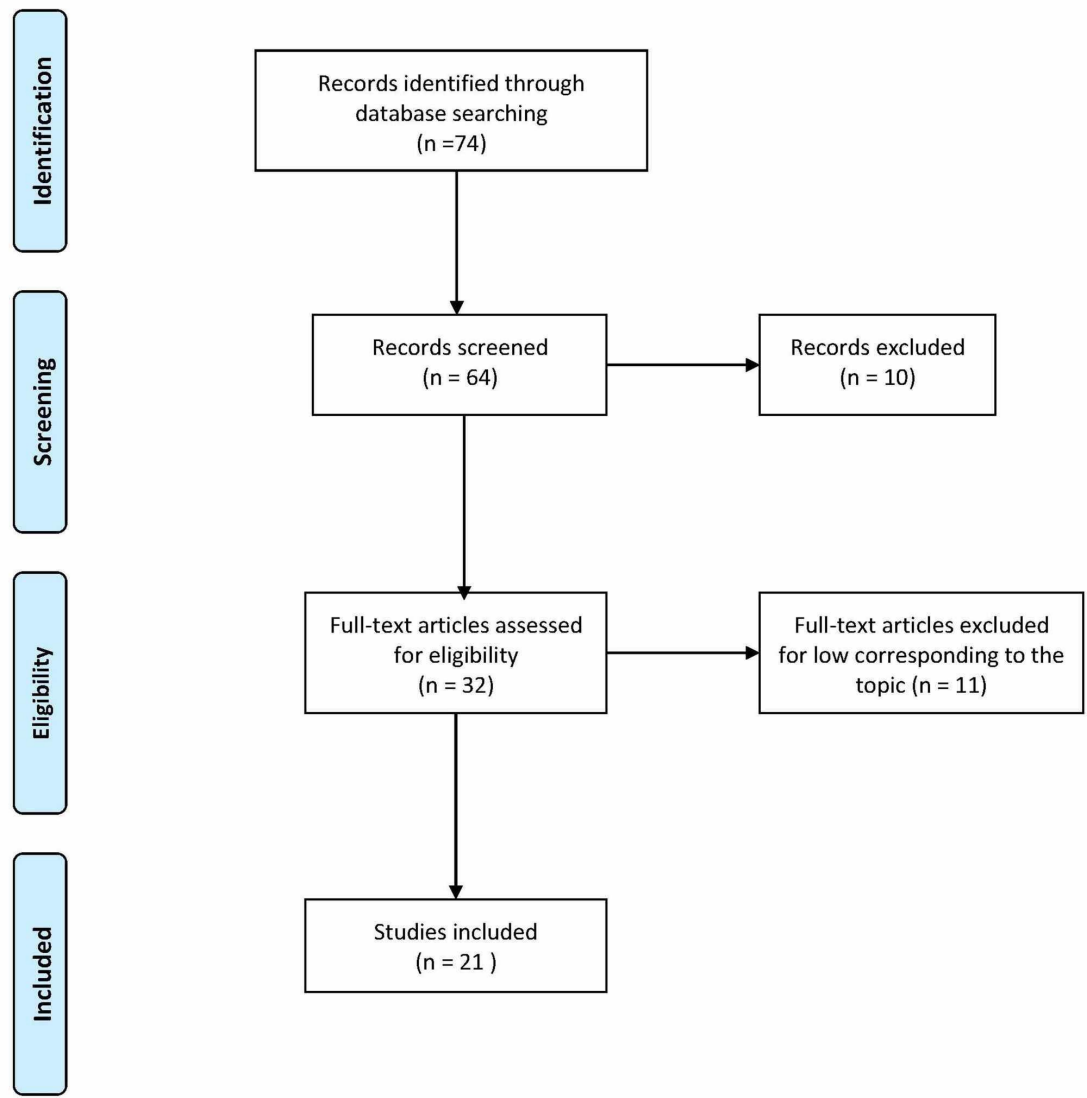
PRISMA, Preferred Reporting Items for Systematic Reviews and Meta-Analyses

The type of studies examined concerns only randomized or non-randomized clinical trials, with or without control group, single or double-blind, since 2005, in order to have a broader overview of the studies carried out in recent years. After making an initial selection by reading the title and abstracts, the potentially eligible studies were identified through a search on the database Pubmed 74 record, and then read in full, evaluating whether or not they should be included in the review (Table 1). The articles useful and relevant to the research question were 21/74. The PRISMA (Preferred Reporting Items for Systematic Reviews and Meta-Analyses) flow chart is in Figure 1.

**Table 1:** Inclusion and Exclusion Criteria

INCLUSION CRITERIA	EXCLUSION CRITERIA
Study design: RCT, parallel and crossover trials. Period: studies from January 2005 to March 2020. Study Format: full text. Population: Users with musculo-skeletal disorders or healthy. Intervention: cervical manipulation. Outcome: improved symptomatology or increased strength.	Study design: Case studies, case series, individual cases, dissertations and conference proceedings. Population: non-musculo-skeletal disorders. Language: Studies included in languages other than English.

Results

The topics covered by the authors relate cervical manipulation to epicondylalgia, cervical pain, temporomandibular joint dysfunction and strength.

Several authors of the selected studies found that the main effect of manipulation is to raise the pain threshold to the pressure that occurs on the sensory tissue corresponding to the manipulated vertebral metamerus (Table 2) [10-14].

**Table 2:** Summary of Studies Examining the Effects of HVLA Techniques HVLA: High velocity low amplitude

AUTHOR - YEAR	INTERVENTION	SAMPLE	METHOD	RESULTS
Oliveira-Campelo et al., 2010	Manipulation group: atlo-occipital joint thrust. Soft tissue group: sub-occipital muscle inhibition technique. Control group: no intervention.	122	Absence of pain, maximum active mouth opening and pressure pain threshold.	The manipulation of the atlo-occipital joint produces an immediate increase in the pain threshold at the pressure of the trigger points on the masseter and temporal muscle and increases the minimum opening of the mouth.
Mansilla-Ferragut et al., 2009	Manipulation group: thrust atlo-occipital joint. Control group: manual therapy	37 women	Absence of pain, maximum active opening of the mouth and pressure pain threshold.	The application of a thrust to the atlo-occipital joint results in an increase in the maximum active opening of the mouth and the pressure pain threshold.
Martinez-Segura et al., 2006	Experimental group: HVLA thrust. Control group: manual mobilization.	70	The assessment was carried out at rest and 5 minutes after treatment.	A single cervical manipulation is more effective than controlled mobilization in reducing resting neck pain and increasing the active range of motion in people with neck pain.
De Camargo et al., 2011	Handling group: thrust C5-C6. Control group: no treatment	37	EMG data of deltoid muscle, increased pain threshold at pressure of upper trapezius muscle, deltoid and C5 spinous process.	Patients in the manipulation group achieved an increase in the pressure pain threshold on both the deltoid and the C5 spinous process, this was not the case on the upper trapezius. At EMG on the manipulation group there was an increase in median frequency at the beginning of the isometric deltoid contraction.
Dunning et al., 2008	Single thrust group on the right C5/C6 zigoapophyseal joint and placebo group	54 asymptomatic	Pre and post C5/6 Thrust using DelSys Surface EMG system.	Immediate increase in EMG activity at rest of the biceps bilaterally, regardless of whether cavitation occurs or not.
Fernández-Carnero et al., 2008	The subjects participated in two experimental sessions in two separate days, at least 48 hours apart. At each session, participants received a randomly assigned manipulative or manual contact intervention.	10	Thermotest system, electronic algometer and dynamometer.	The application of a manipulation to the cervical spine produced an immediate bilateral increase in the pressure pain threshold and reduction in grip pain. There were no significant changes in pain compared to heat/cold and grip strength on the healthy arm.
Botelho and Andrade, 2011	The subjects were randomly divided into two groups: cervical vertebral manipulation and simulated treatment.	18 men (judo athletes)	The force measurements were obtained from a hydraulic dynamometer immediately before and after each intervention.	Gripping force improves after cervical vertebral manipulation.
Ruiz-Sáez et al., 2007	Two groups: 1) manipulative group and 2) placebo group, simulated treatment.	72	A pressure algometer was used to measure the increase in pain threshold at the pressure.	After a single manipulation at C3-C4 level the pressure pain threshold on the latent trigger points of the upper trapezius muscle immediately increases.
García-Pérez-Juana et al., 2018	Subjects were randomly divided into two groups: Group 1: cervical manipulation (right or left), Group 2: fictitious manipulation	54	Immediate outcomes included cervical kinaesthetic sense as evaluated by joint position sensing error (JPSE) and pressure pain thresholds (PPT). At one week, the results of neck pain intensity (numerical pain scale) and neck pain-related disability (Neck Disability Index [NDI]) were also collected.	Mixed model analysis of covariance revealed significant group × time interaction in favor of the cervical thrust manipulation group for JPSE on rotation and extension. There was also significant interaction for PPT changes from C5 to C6 and anterior tibial. At the one-week follow-up, there was a significant interaction for neck-related disability but not for resting neck pain, worse pain or lower pain experienced in the previous week.
Griswold et al., 2018	Compare non-thrust (NTM) and thrust (TM) manipulation on the cervical and thoracic tract for mechanical neck pain	103	The Neck Disability Index (NDI) was the main result. Secondary outcomes included the Patient Specific Functional Scale (PSFS), Numerical Pain Scale (NPRS), Deep Neck Flexural Strength (DCF), Global Assessment of Change (GROC), number of visits and duration of care.	NTM and TM produce equivalent results for patients with mechanical neck pain.
Galindez-Ibarbengoetxea et al., 2018	Comparing the immediate pain effects of a treatment using HVLA manipulation versus single use of a CCF exercise protocol.	25	Measurements included (1) a visual analog scale (VAS) completed during the measurement of range of motion (ROM), (2) an assessment of cervical spine ROM, (3) a pressure pain threshold test (PPT), and (4) electromyographic activation (EMG) of the sternocleidomastoid muscle during a craniocervical flexion test.	Although both interventions were associated with ROM and pain immediately improved after treatment, HVLA manipulation was more effective than CCF exercise in improving ROM and VAS during ROM. None of the interventions led to changes in EMG.
Alonso-Perez et al., 2017	Subjects were randomly assigned to receive: low amplitude high velocity technique (HVLA), joint loosening or cervical lateral glide loosening (CLGM).	75	The pressure pain threshold (PPT) on C7 unilaterally, the trapezius muscle and bilateral lateral epicondyle were measured prior to application of the single MT technique and immediately after application of the MT. Pain catastrophe, depression, anxiety and kinesiophobia were assessed prior to treatment.	The results indicate that hypoalgesia was observed in all groups after treatment in the neck and elbow region (P < 0.05), but loosening induces more hypoalgesic effects. The interaction between catastrophication and HVLA technique suggests that if the level of catastrophication is low or medium, the chances of success are high, but high levels of catastrophication can cause poor results after HVLA intervention.
Bautista-Aguirre et al., 2017	Cervical or thoracic HVLA. The comparison was made with fictitious contact.	88	It was assessed whether there was an influence on the pain threshold at median/ulnar/radial pressure after the execution of the manipulative techniques. Secondary measures included the assessment of painless gripping force.	Manipulation of low cervical and upper thoracic thrust is no more effective than placebo to induce immediate changes in nerve trunk mechanosensitivity of the upper limbs and gripping force in patients with chronic non-specific mechanical neck pain.
Langenfeld et al., 2015	Manual and mechanically assisted manipulations of the thoracic spine compared.	54 participants with acute or chronic neck pain	The primary measure was pain intensity (VAS). Secondary outcome measures are the physical disability of the neck using the Neck Disability Index, the quality of life measured by European Quality of Life Levels 5 Dimension 5 and patient improvement using the global scale of the patient's impression of change.	Both surgeries improve the neck pain. This is a significant result, as manipulation of the thoracic spine for neck pain does not pose the same risk of injury as manipulation of the cervical spine.
Erhardt et al., 2015	The intervention group received high velocity thrust (HVT) at the atlanto-axial segment while the control group was held in the pre-manipulative waiting position.	23 healthy participants	Color flow Doppler ultrasound was used to measure hemodynamics VA3.	HVT in the atlanto-axial joint segment does not affect the hemodynamics of the sub-occipital portion of the vertebral artery during or immediately after HVT in healthy subjects.
Coronado et al., 2015	Three treatments: cervical TM, shoulder TM or shoulder exercise for over two weeks.	78 participants with shoulder pain	The treatments were compared to 25 healthy participants to compare pain sensitivity with that of the clinical baseline participants.	Clinical participants showed increased sensitivity to pain, but did not respond differently to cervical or peripheral TM.
Saavedra-Hernández et al., 2013	One group performed only cervical manipulation and the other the combination of cervical, cervicothoracic and thoracic manipulation.	82 participants with mechanical neck pain	Neck pain intensity, self-reported disability and cervical range of motion were collected at baseline and one week after the intervention of an assessor blinded by patient allocation.	In patients with chronic mechanical neck pain, cervical and thoracic spine manipulation leads to a greater reduction in disability at one week compared to cervical spine manipulation alone, while changes in pain and range of motion are not influenced differently.
Martínez-Segura et al., 2012	Three groups were formed: the first received cervical manipulation on the right, the second on the left and the third thoracic manipulation.	90 participants with bilateral mechanical neck pain	Pressure pain thresholds (PPT) above the C5-6 zygapophyseal joint, lateral epicondyle and anterior tibial muscle, neck pain (11-point numerical pain assessment scale) and cervical spine interval of motion (CROM) were collected at baseline and 10 minutes after surgery by an assessor blinded by patient treatment assignment.	The results of the randomized clinical trial suggest that cervical or thoracic thrust manipulation induces similar changes in PPT, neck pain intensity and CROM.
Dunning et al., 2012	Patients were randomized to receive or manipulate HVLA or mobilization to the upper cervical and thoracic vertebrae.	107 participants with mechanical neck pain	It was evaluated at baseline and after 48 hours: the neck disability index, the numerical pain assessment scale, the flexion rotation test for measuring the C1-2 passive rotation range of motion and the craniocervical flexion test for measuring the motor performance of the deep cervical flexor.	The HVLA group had a greater reduction in pain disability than the mobilization group. In addition, the HVLA group had a significantly greater improvement in both the C1-2 passive rotational movement range and the motor performance of the deep cervical flexor muscles than the group receiving mobilization.
Puentedura et al., 2011	One group received thoracic manipulation and a cervical range-of-motion (ROM) exercise for the first two sessions, followed by a standardized exercise program for another three sessions. The other group received cervical manipulation and the same cervical ROM exercise for the first two sessions and the same exercise program given to the thoracic group for the next three sessions.	24 participants with acute neck pain	The outcome measurements collected one week, four weeks and six months after initiation of treatment included the Neck Disability Index, Numerical Pain Assessment Scale and Fear-Avoidance Beliefs Questionnaire.	Patients who received cervical manipulation demonstrated greater improvements in the Neck Disability Index and Numerical Pain Scale scores at all follow-up times. There was also a statistically significant improvement in the Fear-Avoidance Beliefs Questionnaire physical activity score at all follow-up times for the cervical group.
Fernández-de-las-Peñas et al., 2007	Each subject participated in three experimental sessions on three separate days, at least 48 hours apart. At each session, subjects received the C5-C6 cervical manipulation intervention, placebo or control provided by an experienced therapist.	15 healthy participants	The immediate effect on the pressure pain threshold on the lateral epicondyle of both elbows, both preoperative and 5 minutes post-operative, was assessed.	The application of C5-C6 cervical manipulation produced a greater increase in PPT in both elbows, compared to placebo or control interventions.

Compared to other treatment techniques such as passive mobilization, manual therapy and kinesio taping, vertebral manipulation seems to be more effective [11,13,15,16].

There are no uniform opinions about strength, the results obtained can be influenced by many variables [8,13,17].

Discussion

From the literature examined we have found important insights that describe the effects that can be obtained as a result of cervical manipulation. The effects obtained concerned both the manipulated area and areas not directly connected.

In the study it was noted that a single manipulation on the C5-C6 segment of the spine is able to increase the amplitude of the electromyographic signal and fatigue resistance during a 30-second isometric contraction of a non-spinal muscle (deltoid) innervated by the same spinal segment in patients suffering from cervicalgia [10]. The manipulation produces an excitatory effect on the motor activity of the muscles associated with the upper limb even if they are not anatomically linked to the area of intervention by origin or insertion. This occurs as a result of the neurophysiological effect produced. The duration of this phenomenon cannot be monitored and it occurs independently of the presence of cavitation [16].

In the manipulation of the atlo-occipital vertebra, on the other hand, changes in the degree of active opening of the mouth can be induced, as occurred in the study where, after manipulation, patients were asked to open their mouths to the degree of non-pain and the distance between the upper and lower central incisors was measured, which confirmed the increase compared to the measurement taken at time 0 [11].

In the case of patients with epicondylalgia, thrust and manual therapy techniques were used and the immediate effects were evaluated. The parameters evaluated at the end of treatment were: pressure pain threshold, hot/cold sensitivity and grip pain threshold on the limb affected by epicondylalgia. The grip strength was evaluated on the healthy limb. Subjects were treated twice at a distance of 48 hours. The results suggest that following manipulative intervention, there is a bilateral increase in the pressure pain threshold at the diseased elbow while there were no significant differences in heat/cold sensitivity and grip strength on the healthy side before and after treatment [13].

Cervical manipulation was compared with the use of kinesiotaping in order to determine which therapy was the most effective in reducing cervical pain and improving range of motion. After about a week, the patients in the study were re-evaluated and it was found that only in the group that had undergone the two manipulations, one directed to the central part of the cervical column and the other at the C7-T1 hinge, there had been an increase in the amplitude of movement of the rotation [15].

The study considers manipulative treatment not for therapeutic purposes but for performance [8]. It evaluated whether manipulation could induce an increase in grip strength on judo athletes. Three manipulative interventions were performed on the basis of cervical movement limitations carried out at least 36 hours apart over a period of three weeks. The comparison was made both on the group that received the intervention, comparing the force data before and after the intervention and on the group that received a simulated treatment. The percentage increase in strength at the end of the third intervention was 10.53% on the right hand and 16.82% on the left hand.

The cervical thrust makes changes to the cervical kinesthetic sense, this occurs both on the rotation movement and on the cervical extension. It also produces changes in the pressure pain thresholds (PPTs) of C5 or C6 and also remotely on the anterior tibial muscle. It is possible that the improvements obtained from manipulation are due to an inhibition of neuro-plastic changes within the dorsal horn, influencing the sensitization process.

Therefore, manipulation involves segmental activation of inhibitory pathways that may lead to changes in PPTs even at a distance of the manipulated area. However, in this study, the anterior tibial muscle did not exceed minimal detectable changes (MDC) and for this reason was not considered a reliable result.

As the short-term effects of manipulation were investigated, we can use it to manage pain-related disability and influence the proprioceptive aspect of patients with mechanical neck pain [18]. However, the graded oscillatory technique also appears to be a good alternative that leads to the same results as manipulation in terms of pain, disability and motor performance [19].

Manipulation on the cervical and thoracic spine, combined with joint techniques for the temporomandibular joint, was compared with the protocol of cranial cervical flexion exercises, which consists of 10 repetitions of 10 second contractions interspersed with 10 second rest. No changes in electromyographic activation of the sternocleidomastoid muscle measured at time 0 and 60 seconds after surgery were detected in both groups under study, while improvements in pain evaluated on the visual analog scale (VAS) scale and cervical joint excursion (ROM) were significant. Between the two treatments, the manipulative group showed greater efficacy [20].

Psychological factors may interact with a manual therapy technique in order to induce hypoalgesia. The interaction between catastrophication and the HVLA technique suggests that if the level of catastrophication is low or medium, the chance of success is high, but high levels of catastrophication can cause poor results after HVLA intervention. However, HVLA techniques are able to produce more local hypoalgesia than other manual therapy techniques such as unilateral posterior-anterior loosening or lateral cervical glide loosening [21].

Spinal manipulative therapy seems to cause a hypoalgesic effect even in distal sites [22]. The theory that would explain this phenomenon is defined as "regional interdependence" which claims that a primary disorder may be related to the dysfunction of different regions or systems of the body [23]. Changes in pain perception are also explained on the basis of spinal, supraspinal and peripheral mediated mechanisms [24].

With regard to mechano-sensitivity of the nerve trunks of the upper limbs and gripping force in patients with non-specific chronic mechanical pain in the neck, low cervical or high thoracic manipulation does not induce significant changes [25].

Langenfeld's study compared mechanical and manual manipulation of the thoracic spine to see what kind of influence they had on cervical pain measured with the VAS scale. Both techniques led to a significant result in terms of pain improvement, which allows us to approach cervicalgia by working on an anatomically different district at the site of pain but with interdependent relationships. The use of thoracic manipulation also reduces the risk of any adverse events arising from cervical manipulation [26].

One of the most dangerous adverse events for the patient's health is damage to the vertebrobasilar artery, as well as spontaneous dissection. This is one of the main causes of non-atherosclerotic stroke in young adults. The causality between cervical spinal manipulative treatment and cervical artery dissection has been treated in several studies and so far, there is no evidence that cervical spinal manipulative treatment is causally related to stroke. Most reviews in the literature now report that there is no convincing data to prove or disprove any causality between the two. However, some authors continue to argue the contrary.

Even the manipulative techniques performed on C0-C1, which corresponds to the potentially most dangerous region for the tortuous passage of the vertebral artery, i.e. between C2 and the occiput, do not change the speed of blood flow of the vertebral artery. In Erhardt's study, color flow Doppler ultrasound was used to measure hemodynamics in the sub-occipital portion of the vertebral artery. The systolic peaks and final diastolic rates of three cardiac cycles measured in neutral, pre-high velocity thrust (HVT), post-HVT, post-HVT in neutral position were analyzed. There were no significant differences between the manipulative group and the control group. The study considered healthy subjects [27].

In the management of peripheral pain, spinal manipulative therapy is as effective as other treatments. In shoulder pain, similar effects of pain modulation have been found when a TM stimulus has been applied to the painful end or the painless cervical spine. In addition, these effects did not differ from an active exercise program. The lack of association between pain sensitivity and clinical outcome may suggest non-specific pathways to clinical benefit [22].

In the treatment of a patient suffering from cervicalgia, single manipulation on the cervical region produces the same effects on pain and increased mobility as using multiple manipulations (cervical, cervicothoracic and thoracic). However, multiple manipulation has given better results on the Neck Disability Index, thus having a greater effect on daily life activities [28].

In the past, it was believed that manipulation would bring benefits to the patient related to the biomechanical influence produced by the technique on the vertebral joint. Nowadays, the mechanism of action of the manipulation is explained by the influence of neurophysiological nature so that the manipulation would be able to reduce inflammatory cytokines and increase beta-endorphins [29,30].

According to some authors, thoracic manipulation is as effective as cervical manipulation in terms of PPT, CROM and pain intensity and eliminates, although minimal, the risk of vertebrobasilar artery damage [31]. Conversely, and more recently, in patients with acute neck pain, Puentedura et al. found significantly greater improvements in pain and disability following short- and long-term follow-up when HVLA push manipulation was directed to the cervical spine rather than the thoracic spine; however, the average symptom duration for patients in that trial was only 15 days and the sample size was small (n = 24) [32].

The combination of HVLA thrust manipulation procedures directed at both the upper cervical and upper thoracic joints can improve the overall outcome of patients with mechanical neck pain [33]. In addition, segmental stimulation caused by direct manipulation to the posterior C5-6 vertebral level joint exerts a neural influence on the lateral epicondyle resulting in increased PPT [34].

## Conclusions

A cervical spine thrust manipulation improves cervical kinesthesia sense, pain-related disability and cervical range of motion in participants with chronic mechanical neck pain. Also, significant improvements were found in pain-free handgrip strength increase and pressure pain threshold after cervical HVLA manipulation in patients with lateral epicondylalgia. The manipulation of atlanto-occipital joint produces an immediate increase in pressure pain threshold on trigger points present on the masseter and temporal muscle and increases the degree of active mouth opening.

Concerning strength results in healthy subjects; given the divergent opinions of the authors, we cannot yet state that manipulation can significantly influence this parameter. Better qualitative studies are needed in order to develop more robust evidence of efficacy. Spinal manipulations can also have risks for the patient if applied when not appropriate but the frequency of complications due to vertebral manipulation is very low. Any type of therapeutic intervention has a risk-benefit ratio for the patient and the health professional, considering this relationship, chooses one therapy over another. The manipulation technique has some limitations: patients do not always have good compliance with treatment or sometimes have contraindications. The long-term benefits and frequency of manipulative therapy remain unknown.
